# Supplementary material for: Photovoltaic modules evaluation and dry-season energy yield prediction model for NEM in Malaysia
Source: PLoS One. 2020 Nov 12;15(11):e0241927. doi: 10.1371/journal.pone.0241927 (PMC7660538; doi:10.1371/journal.pone.0241927)
Supplement: Attachment — Submitted filename: Response to Reviewers.pdf [file pone.0241927.s001.pdf]

Response to Reviewers' Comments

The following table states Reviewers' comments vs. our actions.

Comments	Our Actions
**Academic Editor**	
1. Revised or rearranged to clearly demonstrate its novelty specifically the abstract, introduction and conclusion parts of the manuscript.	Abstract, Introduction and Conclusion are revised and stated clearly the novelty and contribution of this work.<br>We have rearranged Introduction section and written a separate paragraph to specify the novelty and contribution of this study (line 22-28). Prior to that, we have summarized all the previous work in terms of region, outcomes, and different from this study in Table 1.<br>Conclusion is revised and added the newly considered parameter, yield factor (suggested by one of the reviewers). The results are compared with the previous research.<br>Reference list is also updated.
2.We suggest you thoroughly copyedit your manuscript for language usage, spelling, and grammar. Suggested by PLOS one: American Journal Experts (AJE)-<br>Upon resubmission, please provide the following:<br>• The name of the colleague or the details of the professional service that edited your manuscript<br>• A copy of your manuscript showing your changes by either highlighting them or using track changes (uploaded as a *supporting information* file)<br>• A clean copy of the edited manuscript (uploaded as the new *manuscript* file)	The whole manuscript has been thoroughly checked for language usage, spelling, and grammar by the two professional colleagues and co-authors of this article.<br>Here are the details:<br><br>**1. Associate Prof Ir Dr Mohammad Lutfi**<br>Advanced Lightning, Power and Energy Research (ALPER),<br>Department of Electrical and Electronic Engineering,<br>Faculty of Engineering,<br>Universiti Putra Malaysia<br>Serdang, Malaysia<br>Email: lutfi@upm.edu.my

	**2. Associate Prof Ir Dr Muhammad Saufi**<br>Head<br>Department of Electrical Engineering<br>Faculty of Electrical and Electronic Engineering (FKEE)<br>Universiti Tun Hussein Onn Malaysia (UTHM)<br>Parit Raja, Johor, Malaysia<br>Email: saufi@uthm.edu.my<br><br>Track changes and edited manuscript have been uploaded.
3.In your Data Availability statement, you have not specified where the minimal data set underlying the results described in your manuscript can be found.<br>Upon re-submitting your revised manuscript, please upload your study's minimal underlying data set as either Supporting Information files or to a stable, public repository and include the relevant URLs, DOIs, or accession numbers within your revised cover letter.	All the data correspond to each figure have been uploaded as *Supporting Information*.
4. We note that one or more of the authors are employed by a commercial company: Radiation Solutions Inc,<br><br>1. Please provide an amended Funding Statement declaring this commercial affiliation, as well as a statement regarding the Role of Funders in your study. If the funding organization did not play a role in the study design, data collection and analysis, decision to publish, or preparation of the manuscript and only provided financial support in the form of authors' salaries and/or research materials, please review your statements relating to the author contributions, and ensure you have specifically and accurately indicated the role(s) that these authors had in your study. You can update author roles in the Author Contributions section of the online submission form.	The commercial Canadian company, Radiation Solutions Inc has no role on the funding. It also does not provide any salaries to the authors. The author has contributed in academic writing and provided guidance in data representation from his personal interest.<br><br>The company has no role in this research. A separate letter of declaration from the author of this company is uploaded in the Supporting Information. If you need any further information, please do not hesitate to contact us.

Please also include the following statement within your amended Funding Statement.<br><br>“The funder provided support in the form of salaries for authors [insert relevant initials], but did not have any additional role in the study design, data collection and analysis, decision to publish, or preparation of the manuscript. The specific roles of these authors are articulated in the ‘author contributions’ section.”<br><br>If your commercial affiliation did play a role in your study, please state and explain this role within your updated Funding Statement.	
5. Please also provide an updated Competing Interests Statement declaring this commercial affiliation along with any other relevant declarations relating to employment, consultancy, patents, products in development, or marketed products, etc.<br><br>Within your Competing Interests Statement, please confirm that this commercial affiliation does not alter your adherence to all PLOS ONE policies on sharing data and materials by including the following statement: "This does not alter our adherence to PLOS ONE policies on sharing data and materials.” (as detailed online in our guide for authors http://journals.plos.org/plosone/s/competing-interests) . If this adherence statement is not accurate and there are restrictions on sharing of data and/or materials, please state these. Please note that we cannot proceed with consideration of your article until this information has been declared.<br><br>Please include both an updated Funding Statement and Competing Interests Statement in your cover letter. We will change the online submission form on your behalf.	The authors have no conflict of interest. The funding matter has been stated in the cover letter.

6. We note you have included a table to which you do not refer in the text of your manuscript. Please ensure that you refer to Table 5 in your text; if accepted, production will need this reference to link the reader to the Table.	Table 5 (now Table 6) is added in the text.

Reviewers' comments	Our Actions
1. Is the manuscript technically sound, and do the data support the conclusions?<br><br>The manuscript must describe a technically sound piece of scientific research with data that supports the conclusions. Experiments must have been conducted rigorously, with appropriate controls, replication, and sample sizes. The conclusions must be drawn appropriately based on the data presented.<br><br>Reviewer #1: Yes<br><br>Reviewer #2: Partly	The conclusion is revised and supports from results are drawn in the conclusion.
2. Has the statistical analysis been performed appropriately and rigorously?<br><br>Reviewer #1: Yes<br><br>Reviewer #2: Yes	No action is taken.
3. Have the authors made all data underlying the findings in their manuscript fully available?<br><br>The PLOS Data policy requires authors to make all data underlying the findings described in their manuscript fully available without restriction, with rare	All the data are made fully available. Data underlying our findings are already presented in respective tables.<br><br>Besides this, data corresponds to each Figure is uploaded separately. All the data in this manuscript are fully available without any restriction.

exception (please refer to the Data Availability Statement in the manuscript PDF file). The data should be provided as part of the manuscript or its supporting information, or deposited to a public repository. For example, in addition to summary statistics, the data points behind means, medians and variance measures should be available. If there are restrictions on publicly sharing data—e.g. participant privacy or use of data from a third party—those must be specified.<br><br>Reviewer #1: Yes<br><br>Reviewer #2: No	
4. Is the manuscript presented in an intelligible fashion and written in standard English?<br><br>PLOS ONE does not copyedit accepted manuscripts, so the language in submitted articles must be clear, correct, and unambiguous. Any typographical or grammatical errors should be corrected at revision, so please note any specific errors here.<br><br>Reviewer #1: No<br><br>Reviewer #2: Yes	Any inconsistency and inaccuracy in the manuscript are checked by the two professional authors. They have thoroughly checked the manuscript and corrected unambiguity and grammatical error at the revised version.
**Reviewer #1:**	
1. The manuscript is reviewed carefully, widely statistical analysis is given in this study for a small period.<br>In my opinion a small period is not enough for showing the differences between types.	We justify of considering small period (5 days) of data in two ways. First, we have reviewed numbers of literatures which states mere difference in the climate of Malaysia all over the year. Second, we have obtained 5 years of data (rainfall and irradiance) from Malaysia Meteorological Department and conducted analysis.<br><br>The location of peninsular Malaysia is near the equator. Due to that, previous researchers

	considered climate of Malaysia as single climate, “tropical hot and humid”. However, we have analyzed meteorological data and categorized the climate of Malaysia into two types of seasons, namely rainy and dry season. Then, we subcategorize the rainy season into maximum and secondary maximum rainfall based on the rainfall data from the meteorological department. This study has considered the dry season which falls on June and July in Klang valley, peninsular Malaysia.<br>During the dry season, the climate is almost similar on most the days. Least rainfall, ambient temperature, and solar irradiance are found to be very similar on most of the days. This is supported by previous researches as well. Researchers, such as Amin et al. mentioned about the predictable weather in Malaysia [1a] from the hot-sunny days experiment. Another research explains the climate of Malaysia as ‘no large variation in temperature’ from the 10 years meteorological data analysis. It shows average solar irradiance in June and July is 4.3 kWh/m^2. It also describes very mere difference in ambient temperature in June (28C) and July (27.7 C) [1b]. We have found another research which states Malaysia as a region characterized as ‘hot and humid all year round’ [1c] and ‘mere distinctive season’ country and its climate is hot and humid [1d][1e].<br><br>Therefore, we obtained rainfall and irradiance data from meteorological department of Malaysia which we have used for seasonal categorization. If reviewers request, we can provide the analysis report.<br>This analysis also supports us to consider 5 days of data to estimate the dry season’s energy yield.<br><br><br>Reference<br>[1a] Amin, N., Lung, C. W., & Sopian, K.

	(2009). A practical field study of various solar cells on their performance in Malaysia. Renewable Energy, 34(8), 1939-1946.<br>[1b] Qahtan, A. M. (2019). Thermal performance of a double-skin façade exposed to direct solar radiation in the tropical climate of Malaysia: A case study. Case Studies in Thermal Engineering, 14, 100419.<br>[1c] Lee, S. W., Lim, C. H., Chan, S. A., & Von, K. L. (2017). Techno-economic evaluation of roof thermal insulation for a hypermarket in equatorial climate: Malaysia. Sustainable cities and society, 35, 209-223.<br>[1d] Heng, S. Y., Asako, Y., Suwa, T., Tan, L. K., Sharifmuddin, N. B., & Kamadinata, J. O. (2019). Performance of a small-scale solar cogeneration system in the equatorial zone of Malaysia. Energy Conversion and Management, 184, 127-138.<br>[1e] Vaka, M., Walvekar, R., Rasheed, A. K., & Khalid, M. (2020). A review on Malaysia's solar energy pathway towards carbon-neutral Malaysia beyond Covid'19 pandemic. Journal of Cleaner Production, 122834.
2. The whole manuscript has to be revised carefully,<br>Some common symbols, for temperature, for efficiency (nu nopt ro) has to be used.	The revised symbols for temperature and efficiency are T and η (eta) respectively. We have revised the whole manuscript including figures.
3. please use c or a for crystalline silicon and amorphous silicon in small letters.	All the changes are done in the manuscript and figures.
4. please check the sentence in line 41 (there is a dimension problem)<br>Also check line 45.	The sentence is checked and the dimension is corrected.
5. It is suggested to combine sentence with previous one (line 67) because a reference is necessary.	The sentence is revised and can be found in the Table 1, which summarizes the literature review. Corrections can be found in Row1.
check and open the sentence on line 68 and explain the reason.	The reason is explained in the Table 1-(Row1, Col4).
6. Please explain which 3 days on line 80.	Explained in the Table 1- (Row2, Col3)
7. It is suggested not to start a new sentence with a ref. number, In X, Table X or Fig. X. but cite them in the sentence.	All the sentences start with a reference are corrected.
8. It is suggested to remove "and environmental" from the heading of section	"and environmental" term is removed and corrected.

2.	
9. It is more convenient to compare the energy rating (kWh/kWp) and it is suggested to write in the manuscript.	The energy rating as yield factor is calculated and compared with past researches. It is included in Table 5.
10. The conc. section is supported by data It is good.	A result on yield factor is included. Appreciate your comments. Thank you.

**Reviewer #2:**	**Our Actions**
1. I suggest reorganizing the abstract, highlighting the novelties introduced, it should contain answers to the following questions:<br>• What problem was studied and why is it important?<br>• What methods were used?<br>• What are the important results?<br>• What conclusions can be drawn from the results?<br>• What is the novelty of the work and where does it go beyond previous efforts in the literature?<br>The originality of the paper needs to be stated clearly.	Abstract is revised based on the suggestion. We have highlighted the novelties, problem and importance of the study, a brief method, and important results. Then conclusion is drawn from the out results.<br><br>In order to highlight the novelty of this study, we have summarized all the previous researches in Table 1 and stated the difference from our study. In a separate paragraph, we have elaborated how this study is novel comparing with the previous efforts conducted in Malaysia and other countries.<br><br>The originality of the paper is also stated clearly in Introduction.
